# Visual Search Behavior During Toileting in Older Patients During the Action-Planning Stage

**DOI:** 10.3390/jfmk10040429

**Published:** 2025-11-05

**Authors:** Lisa Sato, Naoto Noguchi, Munkhbayasgalan Byambadorj, Ken Kondo, Ryoto Akiyama, Bumsuk Lee

**Affiliations:** 1 Doctor’s Program, Graduate School of Health Sciences, Gunma University, Maebashi 371-0044, Japan; satolisa.2014@gmail.com (L.S.); bayasgalan47463@gmail.com (M.B.); 2 Department of Rehabilitation, Harunaso Hospital, Takasaki 370-3347, Japan; 3 Graduate School of Health Sciences, Gunma University, Maebashi 371-0044, Japan; noguchinaoto@gunma-u.ac.jp (N.N.); akiyamaryoto@gunma-u.ac.jp (R.A.); 4 Department of Occupational Therapy, Faculty of Rehabilitation, Gunma Paz University, Takasaki 370-0006, Japan; kenkondoot@gmail.com

**Keywords:** eye-tracking, gaze behavior, visual search, toileting, functional independence, Functional Independence Measure (FIM), older patients

## Abstract

**Background**: Visual search supports action planning and target selection in daily life. Despite toileting being frequent yet high-risk in rehabilitation, gaze analyses specific to toileting remain limited. This study quantified visual search behavior during the approach phase of toileting. **Methods**: Twenty inpatients aged 65 years or older in a convalescent rehabilitation ward participated in the study. At the time of hospital admission, their gaze behavior from toilet room entry to arrival at the bowl was recorded using an eye tracker (Tobii Pro Glasses 2). Moreover, we evaluated a toilet-functional independence measure (toilet-FIM), comprising toileting, toilet transfer, and locomotion at discharge. **Results**: In multiple regression, a longer total gaze time directed towards the toilet seat was associated with a greater toilet-FIM independence (β = 0.446), whereas prolonged gaze to the toilet rim (β = −0.839) and to the right handrail (β = −0.621) were related to lower independence (adjusted R^2^ = 0.715). **Conclusions**: A toilet seat-oriented gaze implies effective action planning for safe sit-down, whereas toilet rim- or handrail-oriented gazes may reflect responses to visual salience or compensatory visual strategies related to reduced independence. These observations could improve our understanding of older patients’ motor planning and spatial perception in toileting.

## 1. Introduction

Visual search behavior is a form of higher-order visual information processing that plays a critical role in the perception of the surrounding environment and in guiding behavior [1]. In everyday activities, people search for task-relevant targets, recognize them as objects of manipulation, and fixate on them for a period of time before initiating movement [2]. Moreover, people allocate more focus to objects relevant to upcoming actions [3,4]. It has been reported that these visual search behaviors reflect sequential cognitive and motor processes and support the execution of goal-directed actions [5,6].

The three-dimensional space that surrounds us is visually perceived as a unified world. However, the brain utilizes an egocentric reference frame to represent three-dimensional space [7], where objects are positioned relative to the individual’s own body, with the horizontal and vertical planes extending from the ego center. The horizontal dimension of space is typically divided into peripersonal and extrapersonal spaces [8,9]. Peripersonal space refers to the space within arm’s reach, where hand and arm movements can be used to directly interact with nearby objects [8,9]. This space is characterized by the integration of visual information with other sensory modalities, such as tactile and proprioceptive inputs. These multisensory signals enable the brain to construct a coherent representation of the body in relation to its surrounding objects [9,10,11]. In this space, visual search behavior primarily serves to dynamically track the spatial relationship between one’s own body and objects in real time, thereby facilitating the integration of visual information with other sensory modalities [12,13].

In contrast, extrapersonal space encompasses the area beyond immediate reach, where direct physical interaction with objects is not possible [8,9]. In this space, visual search behavior primarily serves to monitor the external environment [10,11] and to support spatial navigation and locomotor guidance toward relevant targets [9,14]. In activities involving locomotion followed by object manipulation, visual search supports both spatial navigation and multisensory integration. While visual search in spatial navigation and object manipulation is being widely utilized in daily life tasks, some activities do not benefit from continued visual guidance. Among them, toileting activity is one such representative example. Toileting in Western-style toilets requires sitting backward onto the toilet seat, which typically involves turning one’s back to the toilet seat, moving backward, and adjusting body alignment to achieve appropriate positioning. During this sequence, the toilet seat remains outside the visual field, making it difficult to engage in real-time visual search behavior to guide the action. Previous studies analyzing gaze behavior in these situations have reported that visual search behavior is activated in advance to plan and organize proper body movement [15]. Considering the previous study and the spatial and postural characteristics of toileting activity, it is plausible that people engage in proactive visual search before initiating the toileting activity. However, to date, there has been limited research specifically examining visual search behavior during toileting tasks.

We hypothesized that quantitatively capturing visual search behavior during toileting could provide preliminary insights into the potential underlying mechanisms of action planning processes. In medical and long-term care settings, assistance with toileting is often a critical need. Furthermore, the level of toileting independence has been reported to significantly influence discharge destination and post-stroke patient outcomes [16,17]. Therefore, the aim of this study was to examine visual search behavior during toileting tasks in hospitalized patients and to investigate how visual search at the time of admission relates to toileting independence at discharge.

## 2. Materials and Methods

### 2.1. Participants

Twenty participants using a regular toilet admitted to a convalescent ward, where active activities of daily living (ADL) training for social reintegration after discharge was conducted, were included in this study. The inclusion criteria were as follows: (1) 65 years of age or older, (2) manual wheelchair as a primary source of mobility, (3) being without ocular motility disorder, and (4) having the ability to follow commands. Patients under bladder and bowel management were excluded. Informed consent was obtained from all participants or their families before the study. This study was approved by the ethics committee of the hospital (Approval number: 200103).

### 2.2. Toileting Task

**Figure 1:**
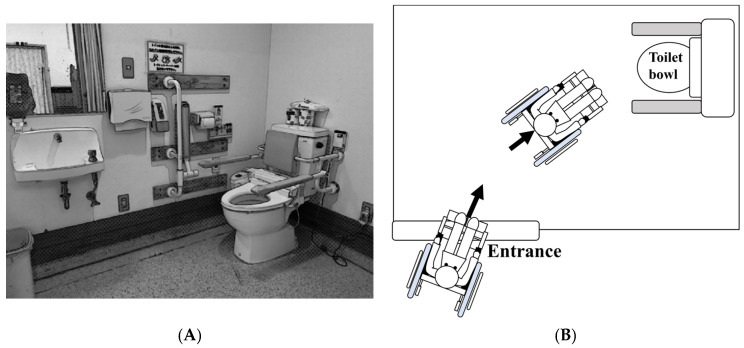
Toilet room environment and typical approach path of a wheelchair user. (**A**) Illustrated photo of the toilet room from a wheelchair user’s perspective at the entrance. (**B**) Typical movement path of a wheelchair user from the entrance to the toilet bowl. The analysis in this study focused on the scope from entrance to the toilet bowl. In this study, analyses targeted the approach interval from entry to arrival at the bowl, prior to any transfer maneuvers.

The measurements were performed in a wheelchair-accessible toilet room (Figure 1). All participants performed the task in the same toilet layout, which was the most common configuration in rehabilitation wards in Japan. The toilet bowl was located on the right-hand side after entering, with a vertical handrail on the right wall and horizontal handrails on both sides of the bowl. Lighting conditions were kept constant, and participants were allowed sufficient time to familiarize themselves with the environment before participating in the study. Figure 1A shows an illustrated photo of the toilet room from a wheelchair user’s eye level at the entrance. Figure 1B illustrates typical movement path of a wheelchair user from the entrance to the toilet bowl. The participants became familiar with the toilet environment and activities before conducting the toileting task. The participants were instructed to perform a series of actions including entering the toilet, excreting, and washing hands, as they would normally do.

### 2.3. Eye-Tracking System

We recorded the patients’ gaze behavior throughout the whole toileting process. An eye-tracking device (Tobii Pro Glasses 2, Tobii Technology Inc., Danderyd, Sweden) was used to measure participants’ visual search behavior during the toileting task. Tobii Pro-Glasses 2 detects eye gaze based on the participants’ field of view. It has a camera with a resolution of 1920 × 1080 at 25 fps and a sampling rate of 50 Hz. The camera has a 90-degree field of view and records videos during toileting with a range of 52 degrees and 82 degrees in the horizontal plane and the vertical plane, respectively. After the measurement was completed, we used the Tobii Pro Lab Software (version 1.171) to analyze the participants’ eye movements.

### 2.4. Eye Movement Measure

**Figure 2:**
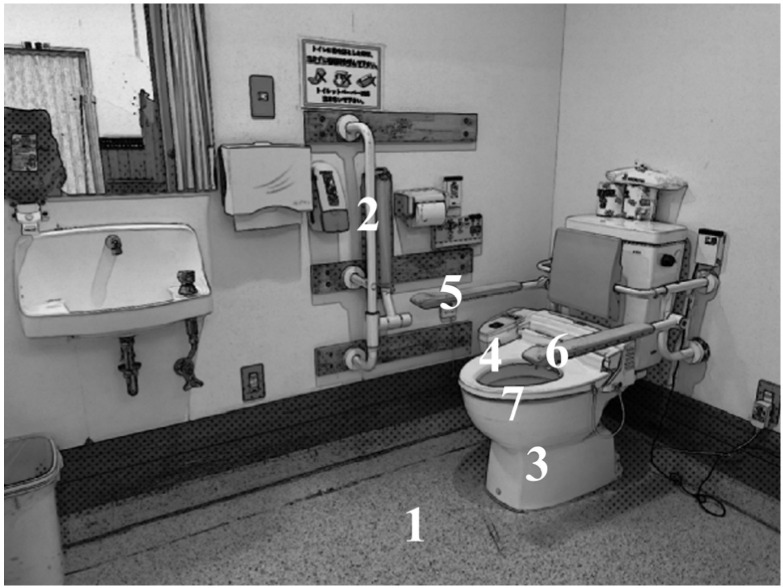
Divided seven AOI regions in the toilet room. 1. Floor, 2. right-wall handrail, 3. toilet bowl (area excluding the seat and handrail), 4. toilet seat (seating surface area), 5. toilet bowl right handrail, 6. toilet bowl left handrail, and 7. toilet seat–bowl border (the border area between the toilet bowl and toilet seat).

The eye movement measure was the total gaze time in each area of interest (AOI). AOIs were selected based on the finding of previous studies [18,19,20,21,22] and our own experience. As the patients gazed at their own bodies before the point of contact, we selected seven AOIs (Figure 2): (1) floor, (2) right-wall handrail, (3) toilet bowl (area excluding the toilet seat and handrail), (4) toilet seat (seating surface area), (5) right-side handrail, (6) left-side handrail, and (7) toilet rim (the border area between the toilet bowl and toilet seat).

We analyzed gaze behavior from toilet room entry to arrival at the toilet bowl (Figure 1B). This entry-to-arrival duration corresponds to the pre-movement planning phase, and therefore could capture two anticipatory gaze modes. The first is just-in-time gaze, occurring approximately zero to one second before action onset [23,24]. The second is anticipatory gaze toward objects that will be manipulated a few seconds later, and the information obtained is used for subsequent motor planning [1]. An executive function study suggested that older adults showed greater difficulty than younger adults in pre-planning complex actions to achieve goals [25]. Based on these reasons, we expected that the total gaze time would be related to toilet-functional independence measure (toilet-FIM: the sum of the FIM scores for toileting, toilet transfer, and locomotion) at discharge.

### 2.5. Clinical Tests

Demographic and clinical characteristics were collected from medical records: age, gender, diagnosis, length of stay in the ward, and discharge destination (home/non-home). Each patient’s basic ADL status was assessed by the FIM [26]. This consists of 13 motor and 5 cognitive items, and the scores range from 1 to 7, with a maximum score of 126 indicating total functional independence of BADLs. The reliability and validity of the FIM have been demonstrated [27].

A hand-grip dynamometer (Tsutsumi seisakusyo, Tokyo, Japan) was used to measure isometric hand-grip strength (kg). The participants were instructed to sit on a chair with their elbows extended and to hold the dynamometer with the lower arm and hand in a neutral position (i.e., 0° supination–pronation and slight extension of hand) [22]. The Berg balance scale (BBS) was used to assess their abilities to safely balance. It contains 14 balance-related tasks, with each step consisting of a five-point ordinal scale ranging from 0 to 4, with 0 indicating the lowest level of activity and 4 the highest level of activity [28]. The mini-mental state exam (MMSE) was used to assess cognitive function. MMSE is a 30-question assessment of cognitive function that evaluates attention and orientation, memory, registration, recall, calculation, language, and ability to draw a complex polygon. It has a maximum score of 30 and a recommended cutoff score of <24 for dementia [29].

### 2.6. Data Collection

We collected the data at two time points. Upon admission to the convalescent ward, the following variables were examined: demographic characteristics, grip strength, BBS, MMSE, and the admission FIM. Upon hospital discharge, the following variables were examined: length of stay in the ward, discharge destination, and the discharge FIM. The assessments were delivered within 2 weeks of admission, and within 1 week prior to discharge.

### 2.7. Statistical Analysis

We tested the assumption of normality of distribution using the Shapiro–Wilk test and found that the data were not normally distributed. As a result, nonparametric statistics were applied. The association between total gazing time for each of the AOI and FIM scores at discharge was examined with Spearman’s rank correlation coefficients. To control for potential Type I error inflation due to multiple testing, Bonferroni correction was applied within each AOI (family-wise correction across six FIM outcomes: total, motor, cognitive, toileting, toilet transfer, and locomotion). Adjusted *p*-values were calculated as *p*_a_*dj* = min [*p* × 6, 1], corresponding to an adjusted significance threshold of *p* < 0.0083. Then, to investigate the contribution of visual search behavior in toileting, a multiple regression analysis was performed. The total gaze time for each AOI, age, gender, grip strength, BBS, and MMSE at admission, toilet-FIM at admission, and diagnosis category were selected as independent variables, and toilet-FIM at discharge as a dependent variable. Analysis was performed by stepwise multiple regression analysis. Additionally, the Mann–Whitney test was used to examine differences in gazing time towards each handrail between later handrail users. The statistical software SPSS ver. 29.0 J for Windows (SPSS Japan, Tokyo, Japan) was used for the analysis. All statistical tests were two-tailed, and the significance level was set at *p* < 0.05.

## 3. Results

Table 1 shows the demographic data and clinical measures of the participants. The median age of the patients was 83.5 (interquartile range (IQR): 73.2–88.7) years with six males and fourteen females.

**Figure 3:**
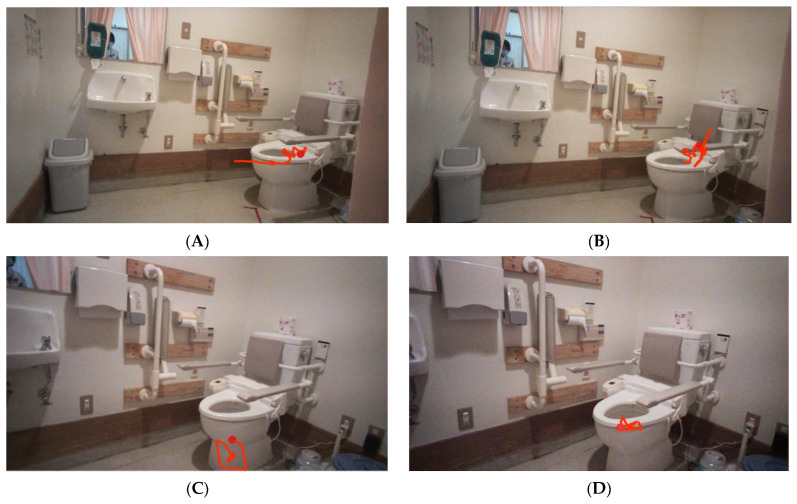
Representative gaze trajectory of one participant within AOIs in the toileting task. The red line indicates the sequence of gaze points, and the red circle indicates the last gaze points. A group of gazing points was fixed on the toilet seat (**A**), the left-side handrail (**B**), the toilet bowl (**C**), and the toilet rim (**D**), respectively.

Figure 3 shows the representative gaze trajectories of one participant within AOIs in the toileting task from toilet room entry to the toilet seat. The red circle indicates the current gaze point, and the red line represents the gaze trajectory leading up to the current point of gazing at the toilet seat (Figure 3A), the left-side handrail (Figure 3B), the toilet bowl (Figure 3C), and the toilet rim (Figure 3D).

Table 2 shows the median and IQR of the total gaze time (ms) for each AOI. Table 3 shows the Spearman’s rank correlations between total gaze time per AOI and FIM outcomes at discharge after Bonferroni correction (within each AOI; family = six FIM outcomes). Bonferroni-adjusted *p*-values were obtained by multiplying each raw *p* by 6, corresponding to an adjusted significance level of *p* < 0.0083. After applying the Bonferroni correction, only one significant correlation remained: total gaze time toward the left-side handrail was negatively correlated with the FIM-Toileting score at discharge (ρ = −0.66 and *p*_a_*dj* < 0.0083).

Multiple regression analysis showed that toilet-FIMs at discharge were associated with total gaze time towards the right-side handrail (β = − 0.621, *p* < 0.001), toilet rim (β = − 0.839, *p* < 0.001), toilet seat (β = 0.446, *p* = 0.019), and BBS score (β = 0.308, *p* = 0.032) on admission. The adjusted R^2^ value was 0.715 (Table 4). Notably, discharge toileting independence appeared to be related to specific visual, physical, and cognitive functions rather than by the overall activity level at admission. Moreover, it is worth noting that there were both positive and negative effects from specific total gazing time on toilet-FIM at discharge. Variance inflation factor (VIF) diagnostics revealed no multicollinearity among the predictors (VIF range: 1.05–1.80). The 95% confidence intervals for significant predictors did not include zero, suggesting stable and precise estimates.

Table 5 shows the comparison of total gaze time towards handrails between later handrail users and non-users. No significant differences in total gaze time towards handrails were found between later handrail users and non-users.

## 4. Discussion

We found that visual search behavior during the action-planning phase of toileting was one of contributing factors in the prediction of toileting independence at discharge. The analysis revealed that a total gaze time on the toilet seat contributed to greater toilet-FIM independence, whereas gazes on both the toilet bowl right handrail and the toilet seat–bowl border were linked to lower independence. Correlation analysis supported these findings by revealing a negative association of toilet-FIM with gazes on the left-side handrail. Based on these search behaviors, we assume that AOI-based visual search behavior could be useful in the analysis of motor planning and spatial perception in daily life tasks.

The key implication of this study is that visual search behavior at the time of admission could serve as an indicator of toileting independence at discharge (Table 4). Previous studies have suggested that ADLs can be explained by higher brain functions such as attention, memory, visuospatial cognition, and executive function [30]. However, to our knowledge, this is the first study to reveal visual search behavior during toileting and identify its relationship with toileting independence. We assume that the patients’ gaze on the toilet seat in the present study may reflect the process of encoding the future contact surface in relation with their body. When turning our back to the toilet seat to sit on it, the toilet seat quickly moves out of the field of vision. Therefore, identifying its spatial relationship with one’s own body needs to be calculated and retained in spatial memory in advance. Such fixations directed to objects required several actions later, called look-ahead fixations, are a form of anticipatory gaze and have been identified in walking and everyday multi-step tasks [31,32]. Additionally, they are thought to support upcoming action sequences. Furthermore, previous research has shown that anticipatory gaze behavior before movement execution is associated with greater movement accuracy and coordination in natural tasks [33]. Taken together, the toilet seat-oriented gaze observed in the present study could be considered a possible anticipatory gaze pattern related to the subsequent sitting action, which may also be associated with greater toileting independence at discharge.

The gaze behaviors observed in this study may also be interpreted in terms of attentional control. Bottom-up attention refers to stimulus-driven processes in which visually salient features, such as high contrast or brightness, attract gaze automatically, whereas top-down attention reflects an intentional, goal-directed allocation of gaze to support upcoming actions [33,34,35]. In addition, some gaze behaviors might also reflect visual compensatory strategies, in which individuals rely more heavily on visual information when proprioceptive or balance control is limited, especially among older adults [36]. These mechanisms are likely to interact dynamically depending on cognitive load and postural demands. Within this framework, a gaze directed to the toilet seat may reflect top-down anticipatory control, while gazes on the toilet rim or handrail could indicate bottom-up or compensatory responses.

Prolonged gaze on the toilet rim, on the other hand, was linked to future lower ADL independence (Table 4). The visuoperceptual feature of the toilet rim in our experimental setting was a high contrast. Contrast sensitivity declines with age, and object recognition performance deteriorates under low-contrast viewing conditions in older adults [37,38]. Previous studies reported that reduced contrast sensitivity can impair balance and dynamic coordination [39], gait [40], and broader ADL performance [41]. In such contexts, visually salient objects tend to attract the gaze reflexively in a bottom-up manner, even when they are not task-relevant [34]. The toilet rim, as a high-contrast cue, may have captured attention automatically, drawing the gaze away from goal-directed targets. Shifting from this stimulus-driven (bottom-up) to a goal-directed (top-down) attentional state requires effortful control over perceptual and cognitive processes [42]. In our older participants, this transition may consume attentional and temporal resources, leaving fewer resources available for predictive gaze control. Such attentional inefficiency could interfere with smooth action planning, compromise movement safety, and ultimately limit toileting independence.

Similarly, prolonged fixation on the right-side handrail was linked to lower toilet-FIM independence at discharge (Table 4). An extended gaze towards the toilet bowl handrail did not necessarily imply that it would later be used for physical support. Rather, such gaze behavior may represent a collision-avoidance strategy when maneuvering within the 50 cm of lateral clearance between the two handrails. Previous studies have shown that age-related declines in proprioceptive and vestibular function can lead to balance impairments, affecting postural control and movement coordination [43]. To stabilize posture, older adults are known to use visual information more than proprioceptive cues [44]. In addition, fixating on a stationary point has also been reported to decrease mediolateral sway [45]. Prolonged gaze on the right-side handrail may indicate a visual compensatory strategy to support postural control when proprioceptive or balance functions are challenged. Although this interpretation is consistent with previous findings linking gaze behavior to sensorimotor planning, it remains hypothetical, as the present study did not directly assess proprioceptive accuracy or postural stability. Future studies combining gaze metrics with proprioceptive or postural assessments are required to confirm this interpretation.

Balance ability, measured by the BBS, was the sole physical function associated with toileting independence. The identification of the BBS score as an independent predictor reaffirms the importance of balance ability in the execution of ADL tasks [46,47,48]. BBS is broadly known to predict FIM outcomes in stroke patients [49] and is also strongly correlated with gait performance in the elderly [12]. Since toileting involves turning, postural transitions, and pulling one’s pants off and on in the limited space, both static and dynamic balance are essential. The present model accounted for 71.5% of the variance in toileting independence, highlighting the value of a comprehensive assessment that includes both higher-order visual information processing and physical factors. These findings may provide preliminary insights that could inform future rehabilitation approaches or evaluation frameworks in toileting support for older adults. Although the present study focused on visuomotor aspects of toileting behavior, it may also be interpreted within an integrative biopsychosocial framework that connects motor planning, cognitive control, and psychophysical well-being across one’s lifespan [50]. This broader perspective emphasizes that functional independence in daily activities arises from the dynamic interaction between perceptual–motor processes and psychosocial factors, highlighting the importance of holistic approaches in understanding and supporting rehabilitation outcomes.

This study has several limitations. Firstly, the investigation was conducted in a single convalescent rehabilitation facility with a relatively small and diagnostically diverse sample, which limits statistical power and generalizability. The high R^2^ value may partly reflect model fitting to the present dataset, and therefore, the regression results should be interpreted with caution. Moreover, because of the limited sample size, disease-specific differences in gaze or motor strategies could not be analyzed. Future research should include a larger and more homogeneous sample to validate the present findings and enable subgroup analyses that clarify potential diagnosis-related differences. Secondly, because the study focused on visual search behavior and postural balance, other potentially influential factors such as contrast sensitivity, attentional function and, executive function were not measured concurrently. Moreover, behavioral aspects such as sitting accuracy, movement hesitation, and postural stability were not assessed following gaze observation, which limited the ability to establish direct evidence linking gaze behavior to movement quality. Future research should employ a comprehensive assessment battery that integrates visual function metrics, including contrast sensitivity with cognitive measures of attention and executive control and behavioral indicators of movement quality, to deepen interpretations of the present results and elucidate the mechanisms underlying action planning during toileting tasks.

## 5. Conclusions

In the present study, we quantified visual search during the pre-movement planning phase of toileting using total gaze time per AOI in patients admitted to a convalescent rehabilitation ward. We found that longer total gaze times directed to the toilet seat and higher BBS at admission were associated with greater toileting independence at discharge, whereas prolonged gazes on the toilet rim and the right-side handrail were related to lower independence. These findings suggest that an anticipatory gaze directed toward the toilet seat may support action planning for safe sitting. Conversely, a gaze toward the rim or handrail might reflect responses to visual salience or compensatory strategies, which could be associated with reduced independence. A quantitative assessment of gaze behavior during toileting may provide useful insights into motor planning and spatial perception, and could serve as a potential tool to support individualized evaluation and rehabilitation planning in clinical settings.

## Figures and Tables

**Table 1:** Demographic data and clinical measures of the patients (*n* = 20).

Demographic	
Age, year, median (IQR)	83.5 (73.2–88.7)
Gender, number, male/female	6/14
Diagnosis, number, (cerebrovascular/musculoskeletal/disuse)	6/9/5
Length of stay in the ward, day, median (IQR)	75.5 (38.7–135.0)
Discharge destination, number, home/non-home	17/3
Clinical measure, median (IQR)	
Grip strength (both hands, kg)	26.5 (22.5–37.0)
BBS, point	34.0 (20.0–45.0)
MMSE, point	24.0 (20.0–26.0)
FIM score, point, median (IQR)	
Admission	
Total	72.5 (60.0–86.0)
Motor	42.0 (35.2–54.7)
Cognitive	30.0 (23.5–32.7)
Toileting	3.0 (1.0–4.7)
Toilet transfer	4.0 (2.2–5.0)
Locomotion	1.5 (1.0–5.0)
Discharge	
Total	111.5 (97.2–117.5)
Motor	80.5 (68.7–84.5)
Cognitive	31.0 (25.2–34.5)
Toileting	6.0 (6.0–7.0)
Toilet transfer	6.0 (6.0–6.0)
Locomotion	6.0 (6.0–6.0)

BBS: Berg balance scale, FIM: functional independence measure, IQR: interquartile range, and MMSE: mini-mental state examination.

**Table 2:** The total gaze time per AOI from entry to toilet bowl arrival.

Variables	Median (IQR)
1. Floor	1210.0 (625.0–4295.0)
2. Right-wall handrail	30.0 (0.0–375.0)
3. Toilet bowl	720.0 (215.0–1220.0)
4. Toilet seat	350.0 (10.0–940.0)
5. Right-side handrail	0.0 (0.0–15.0)
6. Left-side handrail	0.0 (0.0–90.0)
7. Toilet rim	0.0 (0.0–250.0)

AOI: area of interest and IQR: interquartile range (millisecond).

**Table 3:** Spearman’s rank correlations between total gaze time per AOI and FIM outcomes at discharge.

Variables	FIM					
	Total	Motor	Cognitive	Toileting	Toilet Transfer	Locomotion
Total gaze time						
1. Floor	0.43	0.41	0.39	0.32	0.30	0.35
2. Right-wall handrail	0.09	0.03	0.14	0.05	0.39	0.25
3. Toilet bowl	−0.45	−0.55	−0.19	−0.51	−0.39	−0.49
4. Toilet seat	−0.13	−0.18	0.00	−0.18	−0.04	−0.05
5. Right-side handrail	−0.30	−0.31	−0.32	−0.22	−0.17	−0.25
6. Left-side handrail	−0.40	−0.49	−0.15	−0.66 *	−0.46	−0.41
7. Toilet rim	−0.15	−0.20	0.05	−0.22	−0.15	−0.30

FIM: functional independence measure. Bonferroni-adjusted within each AOI: *p*-values were multiplied by 6 (the number of FIM outcomes), corresponding to an adjusted significance level of *p* < 0.0083. The value with an asterisk indicates a correlation significant at the 0.0083 level (two-tailed, Bonferroni-corrected).

**Table 4:** Results of multiple regression analyses of toilet-FIM at discharge.

Dependent Variable	Adjusted R^2^	Independent Variables	B	95% CI for B	β	*p* Value	VIF
Toilet-FIM	0.715	Total gaze time					
		5. Right-side handrail	−1.334	(−1.928, −0.740)	−0.621	<0.001	1.051
		4. Toilet rim	−0.127	(−0.182, −0.072)	−0.839	<0.001	1.793
		7. Toilet seat	0.029	(0.005, 0.053)	0.446	0.019	1.804
		BBS	0.064	(0.006, 0.121)	0.308	0.032	1.061

B: unstandardized regression coefficient, CI: confidence interval, β: standardized regression coefficient, VIF: variance inflation factor, Toilet-FIM: sum score of FIM-Toileting, toilet transfer, and locomotion, and BBS: Berg balance scale. Correlation is significant at the 0.05 level (two-tailed).

**Table 5:** Comparison of total gaze time towards handrails between later handrail users and non-users.

Total Gaze Time	Later Handrail Users Median (IQR)	Later Handrail Non-Users Median (IQR)	*p* Value
2. Right-wall handrail	30 (0.0–1012.5)	180.0 (0.0–405.0)	0.89
5. Right-side handrail	0.0 (0.0–0.0)	0.0 (0.0–20.0)	0.29
6. Left-side handrail	0.0 (0.0–60.0)	0.0 (0.0–120.0)	0.67

IQR: interquartile range (millisecond).

## Data Availability

The data presented in this study are available on request from the corresponding author. The data are not publicly available due to ethical reasons.
